# AN INTRODUCTION TO THE ATHLOS PROJECT: AGEING TRAJECTORIES OF HEALTH: LONGITUDINAL OPPORTUNITIES AND SYNERGIES

**DOI:** 10.1093/geroni/igz038.2940

**Published:** 2019-11-08

**Authors:** Matthew Prina, Demosthenes Panagiotakos, Martin Prince, Martin Bobak, Warren Sanderson, Serguei Scherbov, Jose L Ayuso-Mateos, Jose Maria Haro

**Affiliations:** 1 King’s College London, London, United Kingdom; 2 Harokopio University, Athens, Attiki, Greece; 3 Global Health Institute, King’s College London, London, England, United Kingdom; 4 Department of Epidemiology and Public Health, University College London, London, England, United Kingdom; 5 International Institute for Applied Systems Analysis, World Population Program, Wittgenstein Centre for Demography and Global Human Capital, Laxenburg, Niederosterreich, Austria; 6 Centro de Investigación Biomédica en Red de Salud Mental, Madrid, Madrid, Spain; 7 Parc Sanitari Sant Joan de Déu, Barcelona, Catalonia, Spain

## Abstract

ATHLOS is a 5-year project, funded by the European Union’s Horizon 2020 Research and Innovation Program. Its aim is to achieve a better understanding of healthy ageing, utilising longitudinal data from existing cohort studies. The measure of healthy ageing used within ATHLOS is based on the definition used by the World Health Organization as the ongoing process of developing and maintaining functional ability to enable well-being in older age. The first step of the project was to harmonise 17 community based cohort studies of ageing, covering 38 countries over the world and over 411,000 individuals. In this talk we will discuss the work of the different work packages of the project, including a description of the existing evidence on risk factors of healthy ageing.

